# Three-dimensional ultrasound integrating nomogram and the blood flow image for prostate cancer diagnosis and biopsy: A retrospective study

**DOI:** 10.3389/fonc.2022.994296

**Published:** 2022-10-26

**Authors:** Dong Chen, Yingjie Niu, Haitao Chen, Dequan Liu, Rong Guo, Nan Yao, Zhiyao Li, Xiaomao Luo, Hongyang Li, Shicong Tang

**Affiliations:** ^1^ Department of Ultrasound, The Third Affiliated Hospital of Kunming Medical University, Yunnan Cancer Hospital, Kunming, China; ^2^ Department of Hepatobiliary and Pancreatic Surgery, The Third Affiliated Hospital of Kunming Medical University, Yunnan Cancer Hospital, Kunming, China; ^3^ Department of Breast Surgery, The Third Affiliated Hospital of Kunming Medical University, Yunnan Cancer Hospital, Kunming, China

**Keywords:** prostate cancer, three-dimensional transrectal ultrasound, diagnosis, biopsy, transrectal ultrasound

## Abstract

**Backgrounds:**

Prostate cancer (PCa) is the second most common male cancer in the world and based on its high prevalence and overwhelming effect on patients, more precise diagnostic and therapeutic methods are essential research topics. As such, this study aims to evaluate the value of three-dimensional transrectal ultrasound (3D-TRUS) in the detection, diagnosis and biopsy of PCa, and to provide a basis for clinical practice of PCa.

**Methods:**

Retrospective analysis and comparison of a total of 401 male patients who underwent prostate TRUS in our hospital from 2019 to 2020 were conducted, with all patients having prostate biopsy. Nomogram was used to estimate the probability of different ultrasound signs in diagnosing prostate cancer. The ROC curve was used to estimate the screening and diagnosis rates of 3D-TRUS, MRI and TRUS for prostate cancer.

**Results:**

A total of 401 patients were randomly divided into two groups according to different methods of prostate ultrasonography, namely the TRUS group (251 patients) and the 3D-TRUS group (150 patients). Of these cases, 111 patients in 3D-TRUS group underwent MRI scan. The nomogram further determined the value of 3D-TRUS for prostate cancer. The ROC AUC of prostate cancer detected by TRUS, MRI and 3D-TRUS was 0.5580, 0.6216 and 0.6267 respectively. Biopsy complications were lower in 3D-TRUS group than TRUS group, which was statistically significant (*P*<0.005).

**Conclusions:**

The accuracy of 3D-TRUS was higher in diagnosis and biopsy of prostate cancer. Meanwhile, the positive rate of biopsy could be improved under direct visualization of 3D-TRUS, and the complications could be decreased markedly. Therefore, 3D-TRUS was of high clinical value in diagnosis and biopsy of prostate cancer.

## Introduction

Prostate cancer (PCa) is the second most common male cancer in the world, accounting for about 7.9% of all malignant tumors (1). In Europe and America, the incidence of PCa accounted for the first in masculine malignant tumor and the mortality rate occupied the second. While in China, there were more than 100000 new PCa patients each year (2). A large number of studies had shown that when it occurred to clinical symptoms, most patients had entered the advanced stage (3). Therefore, it is critical to early screen and diagnosis of PCa.

At present, the main clinical screening methods of PCa were serum prostate-specific antigen (PSA), digital rectal examination (DRE), magnetic resonance imaging (MRI) and transrectal ultrasound (TRUS) (4). Studies had found that magnetic resonance imaging (MRI) was an essential part of improving the screening rate of PCa (5) However, there were some limitations of MRI, such as high cost, limited availability, limited use location, etc (6). Ultrasound had been widely used in many medical fields, due to its advantages of being non-invasive, convenient and easy to obtain, and so on (7).

Clinically, the prostate biopsy was considered to be the gold standard for its diagnosis (8). More and more evidence showed that MRI-targeted biopsy can significantly enhance the detection rate of PCa compared with conventional TRUS guided biopsy (9). However, MRI-targeted biopsy could not be performed in all patients, such as patients with implants, pacemakers, or claustrophobia, in addition, MRI-targeted biopsy was expensive and time-consuming (10). Therefore, it was urgent to find a more safe and effective prostate screening method.

At present, studies had shown that the diagnosis of PCa has turned to three-dimensional (3D) information. Nowadays, endoluminal 3D-TRUS was mainly used in the local stage of middle and lower rectal cancer (11). Van der AA et al (12) found that 3D-TRUS guidance or pre-guidance has a higher value in the detection and diagnosis of PCa. However, there were few reports about 3D-TRUS on the diagnosis of prostate disease. This paper aims to study the value of 3D-TRUS in the detection and diagnosis of PCa, thus providing the sound basis for clinical practice.

## Methods

### Patients and ethics

**Figure 1:**
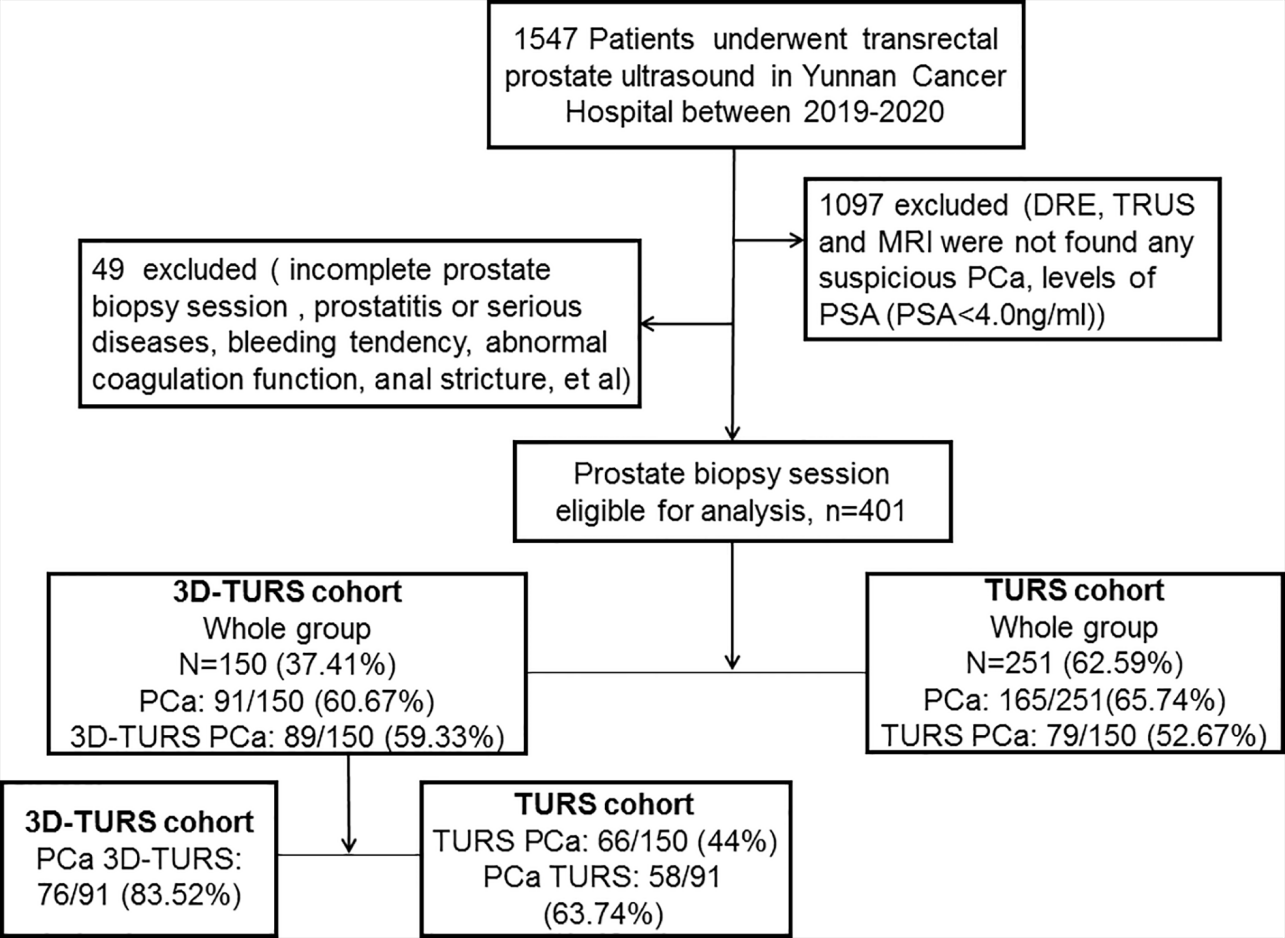
Flow chart for prostatic cancer patients of 3D-TRUS cohort and TRUS cohort.

A total of 1547 male patients who underwent TRUS of the prostate in our hospital from 2019 to 2020 had been retrospectively analysised and compared. Among which, 1097 patients were excluded without indication of biopsy, such as there were no suspicious nodules or lesions were found by DRE, MRI, or TRUS, and PSA was less than 4ng/ml. Among the remaining 450 patients, 13 patients had coagulation abnormalities, 12 patients had active prostatitis, 10 patients had severe diseases, 8 patients had anal stenosis, and 6 patients refused biopsy. Finally, a total of 401 patients were included in the study.TRUS was performed from March 2019 to June 2020, and 3D-TRUS was performed from July 2020 to December 2020, so it was divided into 3D-TRUS group(150 patients) and TRUS group(251 patients).There were 111 patients in the 3D-TRUS cohort were performed MRI (**Figure 1**). The study was approved by the Institutional Review Board of our Hospital (KY201944). Informed consent was obtained from all individual participants included in the study. All procedures implemented in studies involving human participants were under the ethical standards of the institutional and/or national research committee, and with the 1964 Helsinki Declaration and its later amendments or comparable ethical standards.

### Clinical data collection

Retrospective review of medical records and pathology reports of the two cohorts. The variables were statistically analyzed in the two cohorts respectively, including age, height, weight, body mass index (BMI), diabetic,prostate volume, total prostate specific antigen (TPSA), free prostate specific antigen(FPSA), F/T, Gleason score, ISUP group, location, microscopic hematuria, T stage, N stage, M stage.

### Examination method

All 3D-TRUS and TRUS diagnoses of prostate were performed by three senior doctors respectively. Two doctors had more than 5 years of experience in prostate ultrasound diagnosis, and the other doctor had more than 10 years of experience in prostate ultrasound diagnosis. The diagnosis should be made only when two doctors have the same opinion, and reviewed by another doctor with higher seniority. When the two doctors disagree, discuss the diagnosis with another doctor with higher seniority. Denmark BK Pro focus 2202 color Doppler ultrasound diagnostic instrument was used, TRUS: intracavity probe, 3D-TRUS: intracavity 3D probe (model 8838, probe frequency 6, 9, and 12 Mhz). A cleaning enema was performed two hours before the examination. The probe was sleeved to two protective sleeves. The whole prostate was shown after the probe entering the anus slowly. Automatic 3D volume imaging was started to volume imaging for the prostate.

### Puncture biopsy

**Preoperative preparation:** routine evaluation of blood routine test, coagulation function, cardiovascular and cerebrovascular diseases, infection and immune status. Anticoagulant drugs were suspended one week before operation. Antibacterial drugs were used 3 days before operation. Clean enema 2 hours before operation.

**Biopsy position:** The patient adopted the lithotomy position, exposed the anus fully, and disinfected the perineum, anus, and anal canal area with disinfected iodophor.

**Anesthesia:** 3D-TRUS group used subcutaneous anesthesia of perineal surface and peripheral nerve block anesthesia (PPNB), while the TRUS group used subcutaneous anesthesia of perineal surface and subcapsular anesthesia.

**Biopsy process: A** total of 401 patients underwent intraoperative ECG monitoring, and a 20cm 20G biopsy needle was used. In the 3D-TRUS group of 150 patients, if an lesion was partially visible on 3D-TRUS,a 12-core systematic biopsy was performed along with targeted biopsy(12+X),if an lesion was invisible on 3D-TRUS, a 12-core systematic biopsy alone was performed.In the TRUS group of 251 patients,a 12-core systematic biopsy alone was performed,and appropriately increased the number of needles according to the prostate volume to cover most areas of the prostate.All prostate puncture biopsies were conducted independently by 3 doctors with rich prostate puncture experience, and each doctor had more than 200 prostate puncture biopsies experience.During the operation, the VAS score was used to record the pain of the patient. The biopsy specimens were fixed with 10% formaldehyde, and then 3 pathologists with PCa diagnosis experience performed histopathological analysis and Gleason score according to the diagnostic criteria of prostate lesions.

### Follow-up and observation

**Intraoperative complications:** During puncture, complications such as discomfort and hematuria may arise, throughout which, the presence of intraoperative complications was monitored.

**Postoperative complications:** Observe whether the patient had the following complications within one week after surgery: pain, hematuria, infection, urinary tract irritation, and then the symptomatic treatment was a necessity.

### Statistical methods

IBM SPSS statistical software (version 21.0) and GraphPad Prism (version 6.0) were used for statistical analysis. Nomogram was used to estimate the probability of different ultrasound signs in diagnosing PCa. The receiver operating characteristic curve (ROC curve), which was made by Graphpad Prism, had been used to estimate the screening rate and diagnosis rate of 3D-TRUS, MRI, and TRUS for PCa. The enumeration data adopted the chi-square test, and the Fisher exact probability method was adopted when the sample size was less than six. *P*<0.05 was statistically significant.

## Results

### Clinicopathological features of patients

Among 251 patients in the TURS group,166 patients were diagnosed as follicular carcinoma of the prostate, and 85 patients were diagnosed as benign prostatic hyperplasia (BPH) and BPH with chronic inflammation. Among 150 patients in the 3D-TRUS group, 91 patients were diagnosed as follicular carcinoma of the prostate and 59 patients were diagnosed as BPH. The statistical results proved that there were statistical differences in TPSA, FPSA, and N stage between the two (*P*<0.05) (**Table 1**). There were no statistically significant patients who had performed 3D-TRUS and MRI simultaneously in the 3D-TRUS group (*P*>0.05) (**Supplementary Table 1**).

**Table 1:** Clinical and pathological characteristics of prostate cancer patients examined by 3D-TRUS and TRUS.

Variables	Total (n=401)	3D-TRUS (n=150)	TRUS (n=251)	χ^2^	*P*-value
**Age (years)**				0.128	0.720
<60	53	21 (14)	32 (12.75)		
≥60	348	129 (86)	219 (87.25)		
**Height**				1.834	0.176
<167	199	81 (54)	118 (47.01)		
≥167	202	69 (46)	133 (52.99)		
**Weight**				0.037	0.8475
<61	198	75 (50)	123 (49)		
≥61	203	75 (50)	128 (51)		
**BMI**				1.092	0.779
<18.5	34	14 (9.33)	20 (7.97)		
18.5-24	254	97 (64.67)	157 (62.55)		
24-28	95	34 (22.67)	61 (24.3)		
≥28	18	5 (3.33)	13 (5.18)		
**Prostate volume**				0.987	0.321
<51.7	201	80 (53.33)	121 (48.21)		
≥51.7	200	70 (46.67)	130 (51.79)		
**Diabetic**				0.563	0.453
Yes	32	10	22		
No	369	140	229		
**TPSA**				8.986	0.003
≤10	122	59 (39.33)	63 (25.1)		
>10	279	91 (60.67)	188 (74.9)		
**FPSA**				5.025	0.025
≤0.93	78	38 (25.33)	40 (16.13)		
>0.93	320	112 (74.67)	208 (83.87)		
**F/T**				0.001	0.977
<0.25	350	132 (88)	218 (87.9)		
0.25-1.0	48	18 (12)	30 (12.1)		
**Gleason score**				4.745	0.191
≤6 score	18	9 (9.89)	9 (5.62)		
3+4 score	34	11 (12.09)	23 (14.37)		
4+3 score	35	8 (8.79)	27 (16.88)		
≥8 score	164	63 (69.23)	101 (63.12)		
**ISUP group**				7.061	0.133
1	19	9 (9.89)	10 (6.25)		
2	35	11 (12.09)	24 (15.0)		
3	34	8 (8.79)	26 (16.25)		
4	98	43 (47.25)	55 (34.38)		
5	65	20 (21.98)	45 (28.12)		
**Location**				0.427	0.513
One sided	55	22 (24.18)	33 (20.62)		
Two sided	196	69 (75.82)	127 (79.38)		
**Hematuria**				1.417	0.234
Yes	112	35 (25.0)	77 (30.68)		
No	279	105 (75.0)	174 (69.32)		
**T-staging**				3.734	0.292
T1	14	8 (9.2)	6 (3.61)		
T2	64	23 (26.44)	41 (24.7)		
T3	76	24 (27.59)	52 (31.33)		
T4	99	32 (36.78)	67 (40.36)		
**N-staging**				23.058	<0.001
N0	117	37 (43.02)	80 (48.78)		
N1	104	49 (56.98)	55 (33.54)		
N2	29	0 (0)	29 (17.68)		
**M-staging**				0.190	0.663
MO	97	32 (36.36)	65 (39.16)		
M1	137	56 (63.64)	101 (60.84)		

### The value of 3D-TRUS in the diagnosis of PCa

#### The comparison of 3D-TRUS signs between benign and malignant prostate tumors

The following ultrasonic signs were mainly used to diagnose benign and malignant prostate diseases with 3D-TRUS: including echoes, boundary, morphological, capsule, demarcation, fine calcification, and blood flow (13, 14). The common signs of PCa were shown in detail in **Supplementary Figure 1**. The ultrasonic signs had distinct differences between benign and malignant prostate diseases. **Supplementary Figures 1A, B** showed echo signs: Hypoechoic areas could be seen in the peripheral zone of the prostate. **Supplementary Figures 1C, D** showed shape signs: Multiple irregularly shaped hypoechoic areas could be seen in the peripheral zone and inner gland of the prostate. **Supplementary Figures 1E, F** showed boundary signs and abnormal echo areas with unclear boundaries that could be detected in the peripheral zone and inner gland area of the prostate. **Supplementary Figures 1G, H** showed the boundary between the inner and outer glands of the prostate, but the boundary between the inner and outer glands of the prostate was not clear. **Supplementary Figures 1I, J** showed the signs of the capsule, which showed that the capsule of the previous gland was incomplete and part of the capsule was invaded; **Supplementary Figures 1K, L** showed signs of calcification and diffuse distribution of multiple small calcifications in the prostate lesions; **Supplementary Figures 1M, N** showed blood flow signs, and abundant blood flow signals could be seen in the peripheral zone of PCa.

Further statistical analysis of different ultrasonic signs showed that it was significantly higher in patients with PCa (*P*<0.05) (**Table 2**).

**Table 2:** The relationship of signs of 3D-TRUS and detection rate.

Signs of 3D-TRUS	Prostatic cancer	Non prostatic cancer	χ^2^	*P*-value
**Echoes**			81.017	<0.001
Hypoechoic	87 (85.29)	15 (14.71)		
Iso-echo	4 (4.4)	44 (74.58)		
**Boundary**			90.201	<0.001
Clear	7 (12.28)	50 (84.75)		
Unclear	84 (90.32)	9 (9.68)		
**Morphological**			93.595	<0.001
Regular	7 (12.07)	51 (87.93)		
Irregular	84 (91.3)	8 (8.7)		
**Capsule**			63.054	<0.001
Complete	32 (35.16)	59 (64.84)		
Incomplete	59 (100)	0 (0)		
**Demarcation**			54.408	<0.001
Clear	35 (37.63)	58 (62.37)		
Unclear	56 (98.25)	1 (1.75)		
**Fine calcification**			8.457	0.004
Yes	12 (100)	0 (0)		
No	79 (57.25)	59 (42.75)		
**Blood flow**			87.299	<0.001
Rich	77 (95.06)	4 (4.94)		
Not rich	14 (20.29)	55 (79.71)		

Seven ultrasound signs with statistical differences were included in multivariate analysis. The results showed that there were statistical differences in echo, boundary, capsule and fine calcification (**Table 3**).

**Table 3:** Multivariate logistic regression analysis of ultrasonic signs in 3D-TRUS group.

Variables	95% confidence interval	*P*-value
Echoes	0.201-16.646	0.008
Boundary	-0.194-20.677	0.036
Morphological	-18.498-0.000	0.464
Capsule	-0.008-20.442	0.001
Demarcation	-0.316-17.359	0.605
Fine calcification	-12.003-18.897	0.023
Blood flow	-1.386-16.622	0.335

### Predicting the probability of PCa by 3D-TRUS based on nomogram

#### Nomogram development and validation

**Figure 2:**
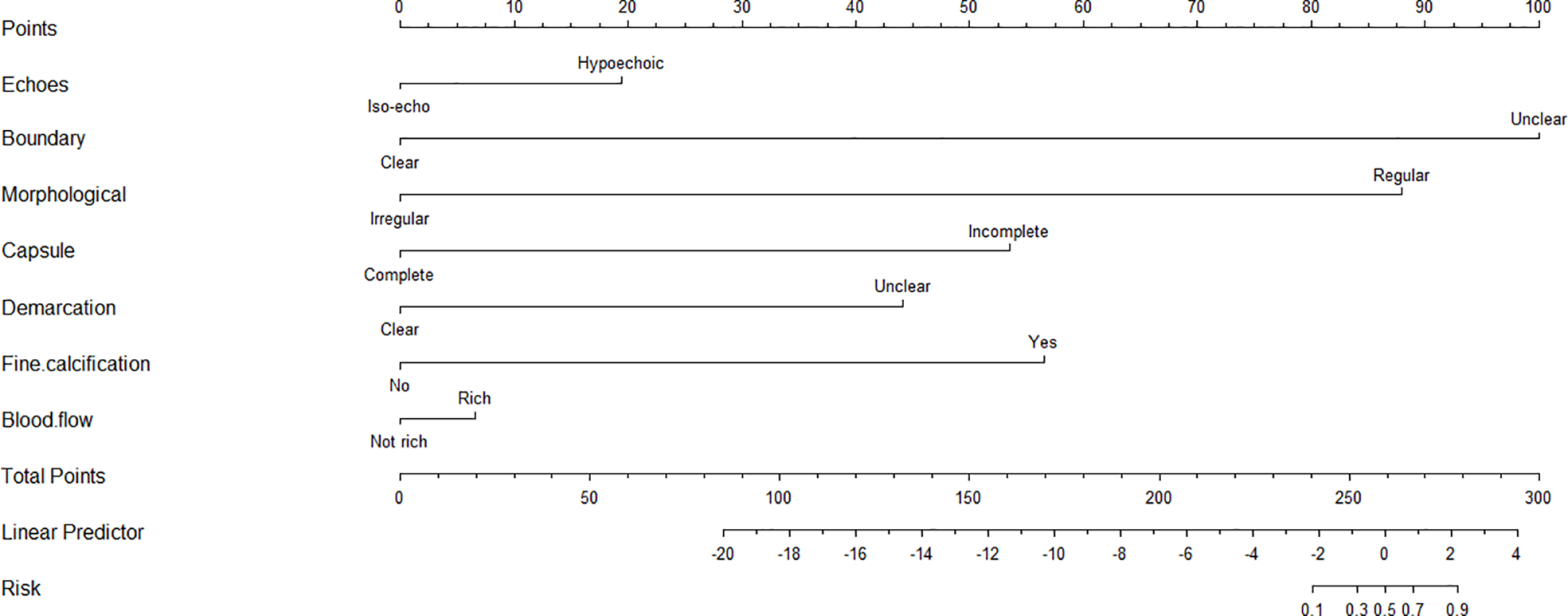
The nomogram for predicting the probability of 3D-TRUS in diagnosis of prostate cancer. To calculate the probability, identify the predictor points on the uppermost point scale that correspond to each patient variable and sum them. The total points projected in the bottom scale indicate the probability of 3D-TRUS in diagnosis of prostate cancer.

**Figure 3:**
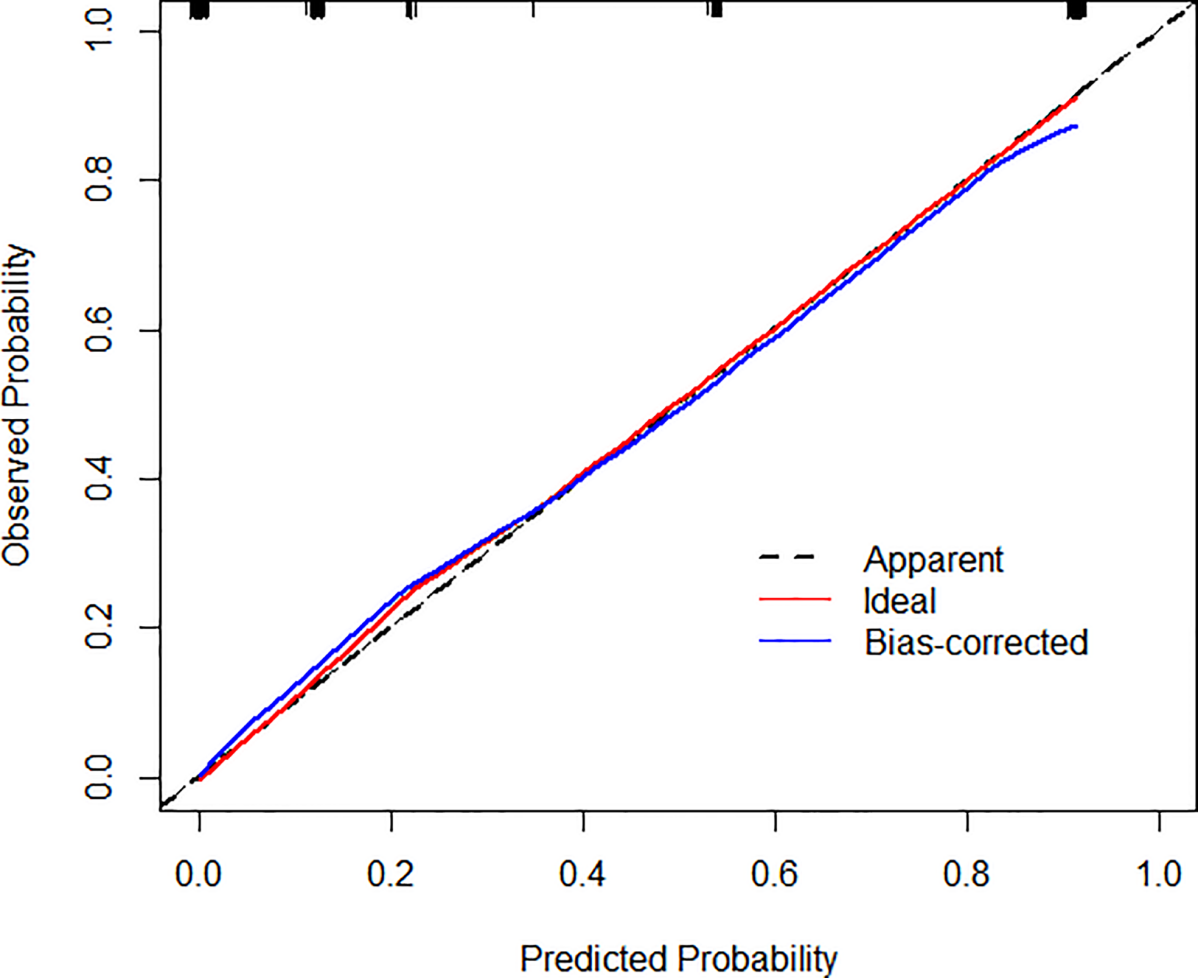
Calibration curves illustrate the observed and predicted diagnostic rate of 3D-TRUS for prostate cancer. The horizontal axis indicates the predicted probabilities measured by the nomogram, and the vertical axis indicates the actual probabilities. For the calibration plot, P = 1.000.

Based on the results of multivariate analysis, echo, boundary, capsule and fine calcification signs were included in the study to form a nomogram to calculate the probability of PCa diagnosis (**Figure 2**). The total score could be used to assign the probability of PCa diagnosis to individual patients using the scale at the bottom of **Figure 2**. Furthermore, the R language was adopted to further verify the formed nomogram. According to the results of the calibration curve, there was no significant difference between the predicted and observed probability of achieving PCa diagnosis, indicating that the line chart is well-calibrated (**Figure 3**).

### The comparison of screening rate and diagnostic rate of 3D-TRUS and TURS in PCa in 3D-TRUS group

#### The comparison of the diagnosis pictures of PCa and non-prostate cancer

**Figure 4:**
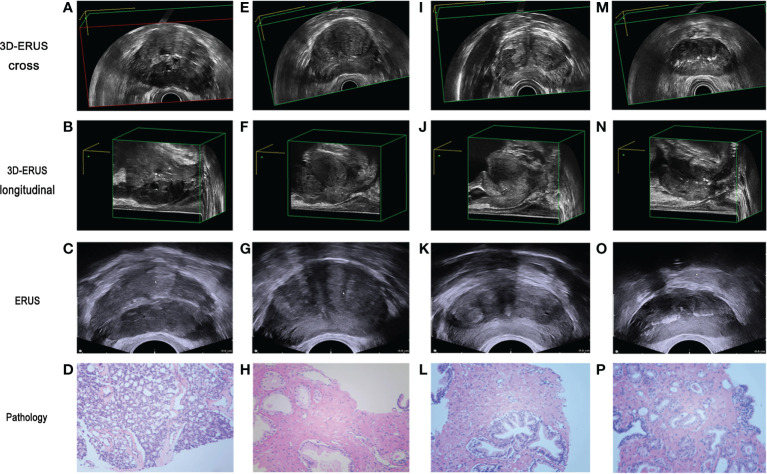
The comparison of prostate cancer and non prostate cancer diagnosis by different diagnostic methods. **(A, B)** 3D-TRUS showed prostate cancer in cross and longitudinal section; **(C)** TRUS showed prostate cancer; **(D)** pathological diagnosis of biopsy samples showed prostate cancer. **(E–F)** 3D-TRUS showed prostate cancer in cross and longitudinal section; **(G)** TRUS showed prostate cancer; **(H)** pathological diagnosis of biopsy samples showed non prostate cancer. **(I–J)** 3D-TRUS showed non prostate cancer in cross and longitudinal section; **(K)** TRUS showed prostate cancer; **(L)** pathological diagnosis of biopsy samples showed non prostate cancer. **(M–N)** 3D-TRUS showed non prostate cancer in cross and longitudinal section; **(O)** TRUS showed non prostate cancer; **(P)** pathological diagnosis of biopsy samples showed prostate cancer.

3D-TRUS could scan the whole prostate from 3D sections: cross-section, longitudinal section, and coronal section. The comparison of different diagnostic methods for the diagnosis of PCa and non-prostate cancer (**Figure 4**).

#### Statistical analysis and comparison of the diagnostic rate of PCa

The 150 patients in the 3D-TRUS group also underwent TRUS examinations at the same time. 91 patients were finally diagnosed with PCa pathologically, which screened out 89 patients (59.33%) with suspected PCa using 3D-TRUS; 66 patients (44.0%) of suspected PCa patients detected by TRUS, there was a statistical difference between the two groups (*P*=0.008) (**Table 4**).

**Table 4:** Detection rate of 3D-TRUS and TRUS in 3D-TRUS group.

	3D-TRUS [n(%)]	TRUS [n(%)]	χ2	*P*-value
Prostatic cancer	89 (59.33%)	66 (44.0%)	7.061	0.008
Non prostatic cancer	61 (40.67%)	84 (56.0%)		

Among the 91 patients with PCa confirmed by pathology, 76 patients (83.52%) were diagnosed by 3D-TRUS, while TRUS diagnosed 58 patients (63.74%). There was a statistical difference between the two groups (*P*<0.002) (**Table 5**).

**Table 5:** Comparison of detection rate of 3D-TRUS and TRUS.

		3D-TRUS	TRUS	χ2	*P*-value
Prostatic cancer	Yes	76 (83.52)	58 (63.74)	9.168	0.002
	No	15 (16.48)	33 (36.26)		

#### Comparison of ROC curve in the diagnosis of PCa

**Figure 5:**
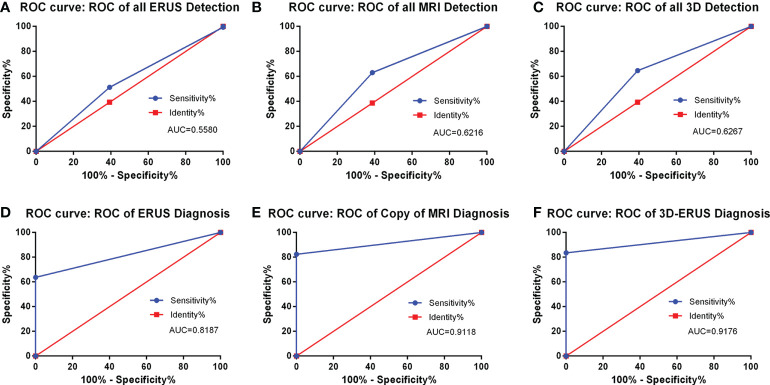
The detection and diagnosis rate of prostate cancer by different diagnostic methods. **(A)**: the ROC curve of all TRUS detection; **(B)** the ROC curve of all MRI detection; **(C)** the ROC curve of all 3D-TRUS detection; **(D)** the ROC curve of TRUS diagnosis; **(E)** the ROC curve of MRI diagnosis; **(F)** the ROC curve of 3D-TRUS diagnosis.

The findings demonstrated that in the screening group, the AUC of PCa detected by TRUS, MRI, and 3D-TRUS were 0.5580, 0.6216, and 0.6267, respectively. Among them, the sensitivity and the specificity were 51.33%, 63.06%, 64.67% and 63.74%, 60.67%, 61.26%, respectively. In the diagnostic group, the AUC of TRUS, MRI, and 3D-TRUS were 0.8187, 0.9118, and 0.9176, respectively. The sensitivity and the specificity were 63.74%, 82.35%, 83.52% and 100, 100, 100%, respectively. The AUC values for 3D-TRUS and MRI were comparable but slightly higher than those for TRUS (*P*<0.05) (**Figure 5**).

### The application of 3D-TRUS in prostate biopsy

#### How to locate the focus with 3D-TRUS

**Figure 6:**
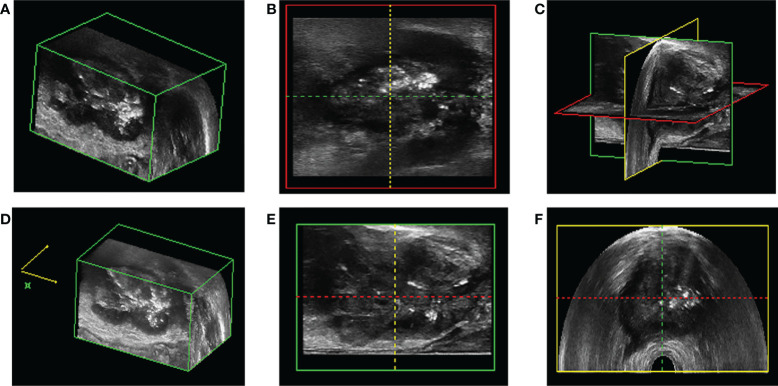
The location of prostate puncture. **(A, D, E)** 3D-TRUS showed prostate cancer in cross section; **(B)** 3D-TRUS showed prostate cancer in coronal section; **(C)** 3D-TRUS showed prostate cancer in three dimensional section; **(F)** 3D-TRUS showed prostate cancer in longitudinal section.

As shown in the **Figure 6**, the location of the focus from the cross-section, longitudinal section, and coronal section could accurately depict the location, size, edge, and outline of the focus under the display of 3D image (**Figure 6**).

#### Blood flow volume image under 3D-TRUS

**Figure 7:**
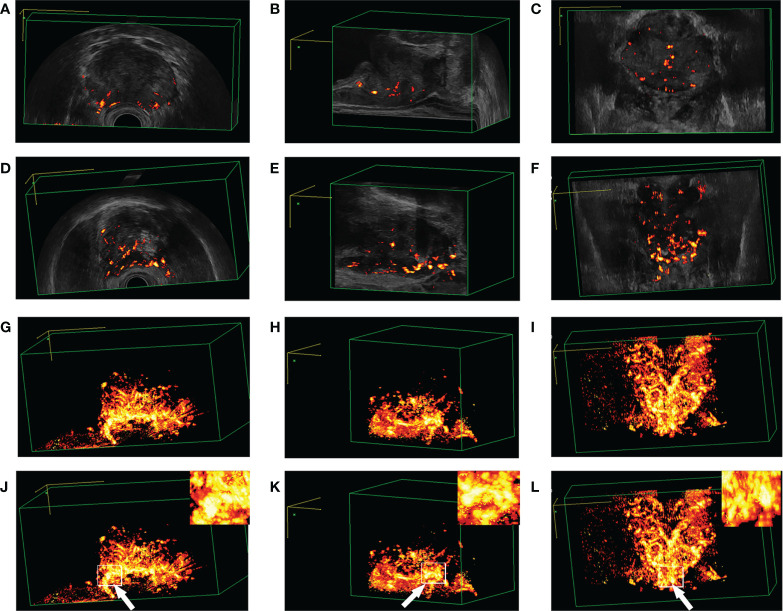
Blood flow plethysmogram of different patients. **(A–C)** Blood flow plethysmogram of normal person; **(D–F)** Blood flow plethysmogram of benign prostatic hyperplasia; **(G–I)** Blood flow plethysmogram of prostate cancer; **(J–L)** the puncture site was the most rich blood flow.

After adjusting the machine parameters, 3D-TRUS could reveal the blood flow of the prostate from a flow volume image based on the location of **Figure 7**. Normal people had nearly the same blood flow as cases with benign prostatic hyperplasia, whereas those with PCa had much more blood flow (**Supplementary Figures 2** and **3**).

#### Selection of prostate puncture location

**Figure 8:**
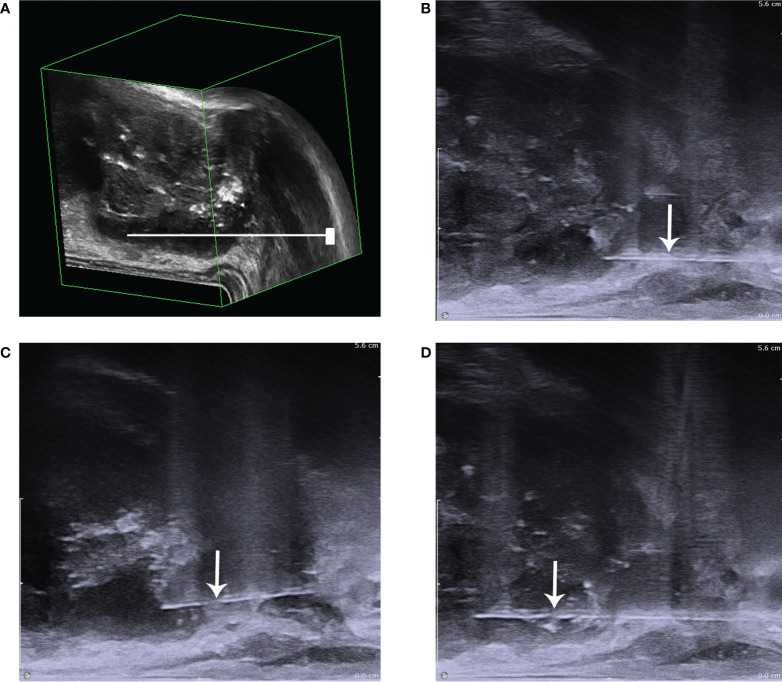
The diagram of prostate puncture. **(A)** The needle is shown to be punctured in the low echo area that has been positioned; **(B)** it showed that before biopsy; **(C)** it showed the process of puncture biopsy,: **(D)** it showed that after biopsy. The white arrow points to the needle.

The prostate puncture was performed under the vision of 3D-TRUS. To improve the accuracy, under the location of **Figure 6**, the blood flow volume image was further used to locate, **Figures 7J–K** further locates the puncture site of the prostate, where the blood supply is most abundant (**Figure 8**).

#### Statistical analysis of the results of prostate biopsy in 3D-TRUS group and TRUS group

In the 3D-TRUS group, 89 patients with suspected PCa were detected before the operation, 84 patients (94.38%) were diagnosed PCa by pathology. In the TRUS group, 96 patients with suspected PCa were detected before the operation, 79 patients (82.29%) were pathologically diagnosed PCa. There was a significant difference in the positive rate of puncturing between the two (*P*=0.011). In the 3D-TRUS group, 61 patients with non-prostate cancer were diagnosed by 3D-TRUS before the operation, of which 7 (13.21%) were PCa; in the TRUS group, 86 patients (55.48%) were diagnosed PCa. There was a statistical difference in the false-negative rate of puncturing between the two (*P*<0.05) (**Tables 6**, **7**).

**Table 6:** Comparison of positive rate of biopsy session between 3D-TRUS and TRUS.

	Prostatic cancer	Non prostatic cancer	χ^2^	95% confidence interval	*P*-value
3D-TRUS -positive	84 (94.38%)	5 (5.62%)	6.443	1.273-10.263	0.011
TRUS -positive	79 (82.29%)	17 (17.71%)			

**Table 7:** Comparison of negative rate of biopsy session between 3D-TRUS and TRUS.

	Prostatic cancer	Non prostatic cancer	χ^2^	95% confidence interval	*P*-value
3D-TRUS –negative	7 (11.48%)	54 (88.52%)	34.578	0.045-0.243	<0.001
TRUS –negative	86 (55.48%)	69 (44.52%)			

#### Relationship between TPSA and positive rate of puncturing in 3D-TRUS group and TRUS group

There was no significant difference between the 3D-TRUS and TRUS groups when TPSA was less than 4 and 4-10 ng/mL (*P*>0.05). However, when TPSA> 10ng/mL, there was a statistical difference in pathological results (*P*<0.05) (**Table 8**).

**Table 8:** The relationship of TPSA and detection rate of prostatic cancer.

TPSA	Prostatic cancer	3D-TRUS	TRUS	χ^2^	*P*-value
<4	Yes	9 (6.0)	5 (1.99)	2.046	0.153
	No	11 (7.33)	16 (6.37)		
4-10	Yes	15 (10.0)	12 (4.78)	0.890	0.345
	No	24 (16.0)	30 (11.95)		
>10	Yes	73 (48.67)	79 31.47)	40.252	<0.001
	No	18 (12.0)	109 (43.43)		

#### The comparison of complications and rebiopsy rate of puncture biopsy in 3D-TRUS group and TRUS group

The results manifested that in the 3D-TRUS group, 7 patients (4.67%) had complications after puncturing. The postoperative complications in the 3D-TRUS group were significantly less than the TRUS group, and there was a significant statistical difference (*P*<0.005) (**Table 9**).In this study, there were 22 diabetic patients in TRUS group and 10 diabetic patients in 3D-TRUS group. No infection complications occurred after puncture biopsy.

**Table 9:** Comparison of complications of biopsy session between 3D-TRUS and TRUS.

	3D-TRUS	TRUS	χ^2^	95% confidence interval	*P*-value
Complication	7 (4.67%)	34 (13.55%)	8.064	0.135-0.724	0.005
Non complication	143 (95.33%)	217 (86.45%)			

Statistical analysis showed that the average number of puncture needles in the two groups were 16.1 and 22.92 respectively (*P*<0.001). In addition, there were 17 patients (6.77%) who had urinary tract irritation in the TRUS group, which was more than 3 patients (2%) in the 3D-TRUS group, there was a significant difference between the two (*P*<0.05) (**Table 10**).

**Table 10:** Comparison of complications after biopsy session between 3D-TRUS and TRUS.

	3D-TRUS	TRUS	χ^2^	*P*-value
Number of puncture needles	16.01	22.92	*-*	<0.001
Bleeding	3 (2%)	12 (4.78%)	2.016	0.156
Non bleeding	147 (98%)	239 (95.22%)		
Infection	1 (0.67%)	1 (0.4%)	1.000	0.712
Non infection	149 (99.33%)	250 (99.6%)		
Pain	0 (0%)	4 (1.59%)	0.302	0.120
Non pain	150 (100%)	247 (98.41%)		
Urinary tract irritation	3 (2%)	17 (50%)	4.514	0.034
Non urinary tract irritation	147	234		

Of all, 1.33% (2/150) of patients in 3D-TRUS group and 2.39% (6/251) of patients in TRUS group underwent re biopsy during the study period. There was no significant difference in the rate of re biopsy between the two groups(*P*>0.05)(**Table 11**).

**Table 11:** Comparison of rebiopsy rate between 3D-ERUS group and ERUS group.

Frequency	3D-ERUS	ERUS	χ^2^	95% confidence interval	*P*-value
First biopsy	147 (98%)	245 (97.61%)	0.065	0.296-4.871	0.798
Rebiopsy	3 (2%)	6 (2.39%)			

#### The comparison of VAS score during puncture biopsy in 3D-TRUS group and TRUS group

According to the VAS score, there were 138 and 125 patients were 0-3 in the 3D-TRUS group and TRUS group respectively, there was a significant statistical difference between the two (*P*<0.05). There were 10 and 75 patients who were 4-6 in the 3D-TRUS group and TRUS group respectively, and 3 and 51 patients were a 7-10 score. There was no significant difference between the two (**Supplementary Table 2**).

## Discussion

Our study was indeed clinically beneficial. Since we used a novel ultrasound examination: 3D-TRUS in this study to screen and diagnose PCa. Moreover, our research was also the first time to compare the pros and cons of 3D-TRUS, TRUS, and MRI in prostate screening and diagnosis, and we found that 3D-TRUS is similar to MRI in screening and diagnosing PCa, which was far superior to TRUS. In addition, we proposed for the first time that under the vision of 3D-TRUS, a lesion-targeted prostate biopsy and blood flow image showing the most abundant blood flow could increase the positive rate of puncturing.

Although TRUS had become an important method of prostate examination now (15), studies had indicated that it has certain limitations in diagnosing PCa, especially for early lesions, whose detection rate was low (16). What’s more, Bai X (17) found that the positive predictive value of TRUS for PCa was 39.38% and that the sensitivity and specificity were 51.41% and 46.90%, which was consistent with our study: the AUC value of TRUS in the screening group was 0.5580, the sensitivity and the specificity were 51.33% and 63.74%. What’s more, the AUC value under the ROC curve of the diagnostic group was 0.8187, the sensitivity was 63.74%, and the specificity was 100%.

Additionally, multi-parameter magnetic resonance imaging (mpMRI) was commonly employed in clinical practice due to its ability to improve PCa detection accuracy. Studies by Yoshizako (18–20) and others showed that compared with simple T2WI, the mpMRI’s detectable rate could be increased from 64% to 79%, the sensitivity was 66-81%, the specificity was as high as 82-92%, and the negative predictive value and positive predictive value were respectively 100%. Ahmed HU (21) et al. found that using mpMRI to examine cases may prevent 27 percent of patients from having a biopsy and reduce the diagnosis of clinically meaningless cancer by 5 percent. There were similar results in our study. In the screening group, the detectable rate of MRI for prostate cancer was 0.6216, the sensitivity was 63.06%, and the specificity was 60.67%. In the diagnosis group, the diagnosis rate was as high as 0.9118, the sensitivity was 82.35%, and the specificity was 100%. What’s more, the diagnosis rate of MRI was as high as 0.9118, the sensitivity was 82.35%, and the specificity was 100%.

The studies of Khanduri S (22) showed that the main signs of TRUS in the diagnosis of PCa are hypoechoic, unclear borders, irregular shapes, incomplete capsules, unclear boundaries between internal and external glands, abundant blood flow, etc. Besides, Steinkohl F (23) et al. revealed that calcification can also be used as an ultrasound sign for the diagnosis of prostate cancer. In this study, 3D-TRUS was used to screen and diagnose PCa, finding that the above seven ultrasonic signs were more clearly displayed on three-dimensional graphics, and there were significant statistical differences in the identification of benign and malignant prostate lesions, which indicated that these seven signs can be used as the diagnostic signs of PCa. Our study detected that the AUC of PCa detected by 3D-TRUS was 0.6267. The AUC values of 3D-TRUS and MRI were similar but higher than TRUS. The AUC of 3D-TRUS in the diagnosis of PCa was 0.9176. The AUC values of 3D-TRUS and MRI were similar and higher than TRUS. Based on these findings, we further used the nomograms to evaluate the diagnostic value of these 4 ultrasound signs for PCa. The formed nomogram may better help doctors diagnose prostate cancer with ultrasound signs. Among them, these had a certain guiding significance for the diagnosis of PCa.

The gold standard for the diagnosis of prostate cancer was an ultrasound-guided biopsy. Birs A (24) and other studies had shown that the MRI-TRUS fusion technique has a very high biopsy rate for PCa. However, fusion biopsies required a longer learning curve (25, 26). In addition, MRI had some disadvantages, such as high economic cost, long examination time, image which has artifacts, differences caused by different observers, and not applicable to all patients (27, 28). In this study, 3 experienced ultrasound doctors selected the above 7 ultrasound signs for 3D-TRUS ultrasound localization, which could roughly locate the suspected lesions. Further use of photoplethysmography [PPG) for localization (consistent with the results of the NelsonED study (29)] showed that the sites with the most abundant blood flow coincided with the suspected lesions previously located, and then punctured the located sites under direct vision. Our study showed that there was a significant difference in the positive rate of puncturing between the 3D-TRUS group and the TRUS group (*P*=0.011). And the false-negative rate of the 3D-TRUS group was much lower than that of the TRUS group. There was a significant statistical difference between the 3D-TRUS group and the TRUS group (*P* < 0.05). In addition, we found that when TPSA<4 or TPS was 4-10ng/mL there was no significant difference in the puncture positive rate between the 3D-TRUS group and TRUS group, but when TPSA > 10ng/mL, there was a significant difference in the puncture positive rate between the two groups (*P* < 0.05). The results proved that with the increase of TPSA, 3D-TRUS could detect more malignant lesions of the prostate.

In addition, we further compared the study of complications after prostate puncture biopsy. The results showed that the postoperative complications in the 3D-TRUS group were significantly less than those in the TRUS group. Further to a subgroup analysis of postoperative complications showed that urinary tract irritation in the TRUS group was higher than that in the 3D-TRUS group, which may be related to the difference in the number of puncture needles between the two groups, and the number of puncture needles in the TRUS group was significantly higher than in the 3D-TRUS group, indicating that 3D-TRUS-guided targeted puncture could significantly reduce complications after biopsy. In this study, both TRUS group and 3D-TRUS group were prevented from using antibiotics before operation, and only one patient in each group had infection complications after operation. Preoperative prophylactic use of antibiotics can better control the incidence of postoperative infection complications. In this study, diabetic patients suffered from postoperative infection complications due to preoperative prophylactic use of antibiotics.Study reported (30) that the re-biopsy rate of patients with traditional transrectal prostate biopsy was three times higher than that of transperineal prostate system biopsy (15.4% Vs 5.26%), and targeted biopsy could further reduce the re-biopsy rate. In this study, the re biopsy rates of patients in the 3D-TRUS group and TRUS group were 1.33% and 2.39%, slightly lower than the previous study results. The main reason was that the 3D-TRUS group combined systematic puncture and targeted puncture, and the TRUS group combined systematic puncture and saturated puncture.What’s more, the tolerance of patients was good, thus it can be applied to clinical practice.

At present, prostate biopsy was still the gold standard for the diagnosis of PCa, but there was a certain rate of missed diagnosis in puncture biopsy, and there were also complications after puncture. Meissner and Louise et al. (31, 32)recently reported that among patients highly suspected of PCa in MPMRI and PSMA-PET, patients with strong uptake of PSMA and MRI positive could avoid definitive biopsy and directly conduct definitive treatment. Avoiding prostate biopsy before RP might be an effective choice. Avoiding biopsy and direct surgical treatment could enhance patient tolerance, reduce complications (including psychological burden and anxiety) after puncture and reduce waiting time for diagnosis, which might be applied to clinical practice after further research in the future.

In summary, we believe that our research has significant clinical implications and demonstrates the critical importance of 3D-TRUS in prostate cancer screening and diagnosis. However, this article had several limitations: this study was a retrospective study involving a limited number of patients in an institution. The nomogram had not been validated by large, independent, external, or forward-looking cohorts. In addition, due to the different anesthesia methods between the two groups, it might affect the pain score during puncture and postoperative complications.This study only compared the imaging manifestations of TRUS, 3D-TRUS and MRI of prostate follicular carcinoma. Because of the low incidence of other special types of prostate cancer, it was not included in this study for comparison of imaging characteristics. However, 3D-TRUS was still recommended for clinical screening and diagnosis of PCa.

## Data availability statement

The original contributions presented in the study are included in the article/**Supplementary Material**. Further inquiries can be directed to the corresponding authors.

## Ethics statement

The studies involving human participants were reviewed and approved by Institutional Review Board of Yunnan Cancer Hospital. The patients/participants provided their written informed consent to participate in this study. Written informed consent was obtained from the individual(s) for the publication of any potentially identifiable images or data included in this article.

## Author contributions

Conception and design: ST. Administrative support: XL, HL, DL. Provision of study materials or patients: DC, YN. Collection and assembly of data: HC. Data analysis and interpretation: DC, YN, ST. Manuscript writing: All authors. All authors contributed to the article and approved the submitted version.

## Funding

The present study was supported by the National Natural Science Foundation of China (grant no. 81960542 and 81960517), Science and Technology Project of Yunnan Provincial Science and Technology Department (grant no.202001AU070053 and 202001AU070093), Scientific Research Foundation of Yunnan Education Department (grant no. 2019J1288 and 2020J0198). Yunnan Health Training Project of High Level Talents (grant no. H-2019075).

## Conflict of interest

The authors declare that the research was conducted in the absence of any commercial or financial relationships that could be construed as a potential conflict of interest.

## Publisher’s note

All claims expressed in this article are solely those of the authors and do not necessarily represent those of their affiliated organizations, or those of the publisher, the editors and the reviewers. Any product that may be evaluated in this article, or claim that may be made by its manufacturer, is not guaranteed or endorsed by the publisher.

## Supplementary material

Supplementary Figure 1 The common ultrasonic signs in the diagnosis of prostate cancer. **(A, B)** show echoes signs: hypoechoic areas can be seen in the peripheral zone of the prostate. **(C, D)** show morphological signs: multiple irregularly shaped hypoechoic areas can be seen in the peripheral zone and inner gland of the prostate. **(E, F)** show boundary signs, and abnormal echo areas with unclear boundaries can be seen in the peripheral zone and inner gland area of the prostate. **(G, H)** show the demarcation signs, the boundary between the inner and outer glands of prostate cancer is not clear. **(I, J)** show capsule signs, which shows that the capsule of the previous gland is incomplete and part of the capsule is invaded; **(K, L)** show signs of fine calcification, diffuse distribution of multiple fine calcifications in prostate lesions; **(M, N)** show blood flow signs, and abundant blood flow signals can be seen in the peripheral zone of PCa.

Supplementary Figure 2 Blood flow plethysmogram of normal person. **(A)** Blood flow plethysmogram of normal person in cross section; **(B)** Blood flow plethysmogram of normal person in longitudinal section; **(C)** Blood flow plethysmogram of normal person in coronal section; **(D)** Blood flow chart of normal person in cross section; **(E)** Blood flow chart of normal person in longitudinal section; **(F)** Blood flow chart of normal person in coronal section.

Supplementary Figure 3 Blood flow plethysmogram of benign prostatic hyperplasia. **(A)** Blood flow plethysmogram of benign prostatic hyperplasia in cross section; **(B)** Blood flow plethysmogram of benign prostatic hyperplasia in longitudinal section; **(C)** Blood flow plethysmogram of benign prostatic hyperplasia in coronal section; **(D)** Blood flow chart of benign prostatic hyperplasia in cross section; **(E)** Blood flow chart of benign prostatic hyperplasia in longitudinal section; **(F)** Blood flow chart of benign prostatic hyperplasia in coronal section.
