# Supplementary material for: Three-dimensional ultrasound integrating nomogram and the blood flow image for prostate cancer diagnosis and biopsy: A retrospective study
Source: Front Oncol. 2022 Oct 26;12:994296. doi: 10.3389/fonc.2022.994296 (PMC9641235; doi:10.3389/fonc.2022.994296)
Supplement: Supplementary file 1 [file Table_1.docx]

**Supplementary Table1 Clinical and pathological characteristics of prostate cancer patients examined by 3D-ERUS and MRI**

| **Variables** | **Total (n=261)** | **3D-ERUS((n=150)** | **MRI**  **(n=111)** | **χ^2^** | **P-value** |
| --- | --- | --- | --- | --- | --- |
| **Age(years)** |  |  |  | 0.009 | 0.924 |
| <60 | 37 | 21(14) | 16(14.41) |  |  |
| ≥60 | 224 | 129(86) | 95(85.59) |  |  |
| **Height** |  |  |  | 0.023 | 0.878 |
| <167 | 142 | 81(54) | 61(54.95) |  |  |
| ≥167 | 119 | 69(46) | 50(45.05) |  |  |
| **Weight** |  |  |  | 0.047 | 0.829 |
| <61 | 129 | 75(50) | 54(48.65) |  |  |
| ≥61 | 132 | 75(50) | 57(51.35) |  |  |
| **BMI** |  |  |  | 0.576 | 0.902 |
| <18.5 | 23 | 14(9.33) | 9(8.11) |  |  |
| 18.5-24 | 166 | 97(64.67) | 69(62.16) |  |  |
| 24-28 | 62 | 34(22.67) | 28(25.23) |  |  |
| ≥28 | 10 | 5(3.33) | 5(4.5) |  |  |
| **Prostate volume** |  |  |  | 0.212 | 0.645 |
| <51.7 | 136 | 80(53.33) | 56(50.45) |  |  |
| ≥51.7 | 125 | 70(46.67) | 55(49.55) |  |  |
| **TPSA** |  |  |  | 0.039 | 0.844 |
| ≤10 | 104 | 59(39.33) | 45(40.54) |  |  |
| ＞10 | 157 | 91(60.67) | 66(59.46) |  |  |
| **FPSA** |  |  |  | 0.000 | 0.984 |
| ≤0.93 | 66 | 38(25.33) | 28(25.23) |  |  |
| ＞0.93 | 195 | 112(74.67) | 83(74.77) |  |  |
| **F/T** |  |  |  | 0.022 | 0.881 |
| ＜0.25 | 229 | 132(88) | 97(87.39) |  |  |
| 0.25-1.0 | 32 | 18(12) | 14(12.61) |  |  |
| **Gleason score** |  |  |  | 0.824 | 0.844 |
| ≤6 score | 18 | 9(9.89) | 9(13.24) |  |  |
| 3+4 score | 19 | 11(12.09) | 8(11.76) |  |  |
| 4+3 score | 12 | 8(8.79) | 4(5.88) |  |  |
| ≥8 score | 110 | 63(69.23) | 47(69.12) |  |  |
| **ISUP** **group** |  |  |  | 1.039 | 0.904 |
| 1 | 18 | 9(9.89) | 9(13.24) |  |  |
| 2 | 19 | 11(12.09) | 8(11.76) |  |  |
| 3 | 12 | 8(8.79) | 4(5.88) |  |  |
| 4 | 77 | 43(47.25) | 34(50.0) |  |  |
| 5 | 33 | 20(21.98) | 13(19.12) |  |  |
| **Location** |  |  |  | 0.109 | 0.741 |
| One sided | 40 | 22(24.18) | 18(26.47) |  |  |
| Two sided | 119 | 69(75.82) | 50(73.63) |  |  |
| **Hematuria** |  |  |  | 0.002 | 0.967 |
| Yes | 63 | 35(25.0) | 28(25.23) |  |  |
| No | 188 | 105(75.0) | 83(74.77 |  |  |
| **T-staging** |  |  |  | 0.240 | 0.971 |
| T1 | 14 | 8(9.2) | 6(8.96) |  |  |
| T2 | 43 | 23(26.44) | 20(29.85) |  |  |
| T3 | 41 | 2427.59) | 17(25.37) |  |  |
| T4 | 56 | 32(36.78) | 24(35.82) |  |  |
| **N-staging** |  |  |  | 0.090 | 0.765 |
| N0 | 67 | 37(43.02) | 30(45.45) |  |  |
| N1 | 85 | 49(56.98) | 36(54.55) |  |  |
| N2 | 0 | 0(0) | 0(0) |  |  |
| **M-staging** |  |  |  | 0.330 | 0.566 |
| M_O_ | 59 | 32(36.36) | 27(40.91) |  |  |
| M_1_ | 95 | 56(63.64) | 39(59.09) |  |  |
