# Supplementary material for: Three-dimensional ultrasound integrating nomogram and the blood flow image for prostate cancer diagnosis and biopsy: A retrospective study
Source: Front Oncol. 2022 Oct 26;12:994296. doi: 10.3389/fonc.2022.994296 (PMC9641235; doi:10.3389/fonc.2022.994296)
Supplement: Supplementary file 2 [file Table_2.docx]

**Supplementary table 2 VAS score in 3D-ERUS and ERUS cohorts**

| VAS | Prostatic cancer | 3D-ERUS | ERUS | χ^2^ | *P*-value |
| --- | --- | --- | --- | --- | --- |
| 0-3 | Yes | 83(54.97) | 50(19.92) | 10.648 | 0.001 |
|  | No | 55(36.42) | 75(29.88) |  |  |
| 4-6 | Yes | 6(3.97) | 30(11.95) | 1.446 | 0.229 |
|  | No | 4(2.65) | 45(17.93) |  |  |
| 7-10 | Yes | 1(0.66) | 16(6.37) | 0.005 | 0.943 |
|  | No | 2(1.32) | 35(13.94) |  |  |
