# Supplementary figures and images for: Three-dimensional ultrasound integrating nomogram and the blood flow image for prostate cancer diagnosis and biopsy: A retrospective study
Source: Front Oncol. 2022 Oct 26;12:994296. doi: 10.3389/fonc.2022.994296 (PMC9641235; doi:10.3389/fonc.2022.994296)

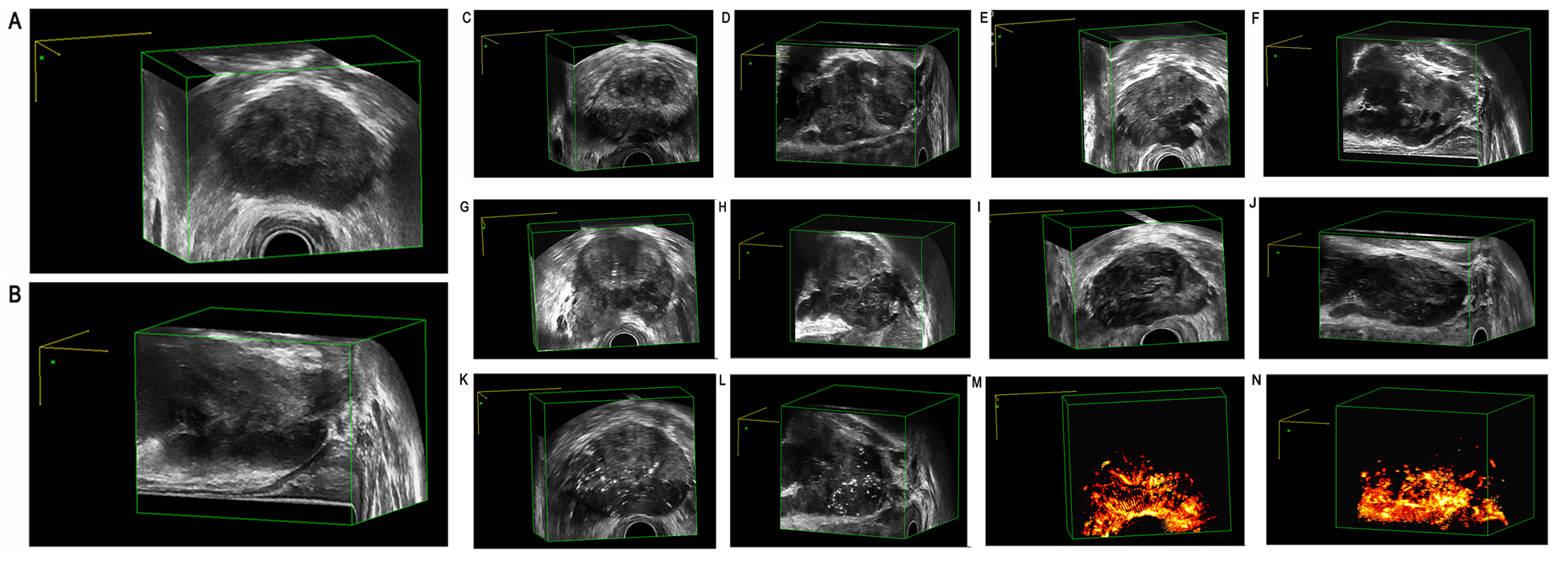

Supplement: Supplementary Figure 1 — The common ultrasonic signs in the diagnosis of prostate cancer. (A, B) show echoes signs: hypoechoic areas can be seen in the peripheral zone of the prostate. (C, D) show morphological signs: multiple irregularly shaped hypoechoic areas can be seen in the peripheral zone and inner gland of the prostate. (E, F) show boundary signs, and abnormal echo areas with unclear boundaries can be seen in the peripheral zone and inner gland area of the prostate. (G, H) show the demarcation signs, the boundary between the inner and outer glands of prostate cancer is not clear. (I, J) show capsule signs, which shows that the capsule of the previous gland is incomplete and part of the capsule is invaded; (K, L) show signs of fine calcification, diffuse distribution of multiple fine calcifications in prostate lesions; (M, N) show blood flow signs, and abundant blood flow signals can be seen in the peripheral zone of PCa. [file Image_1.tiff]

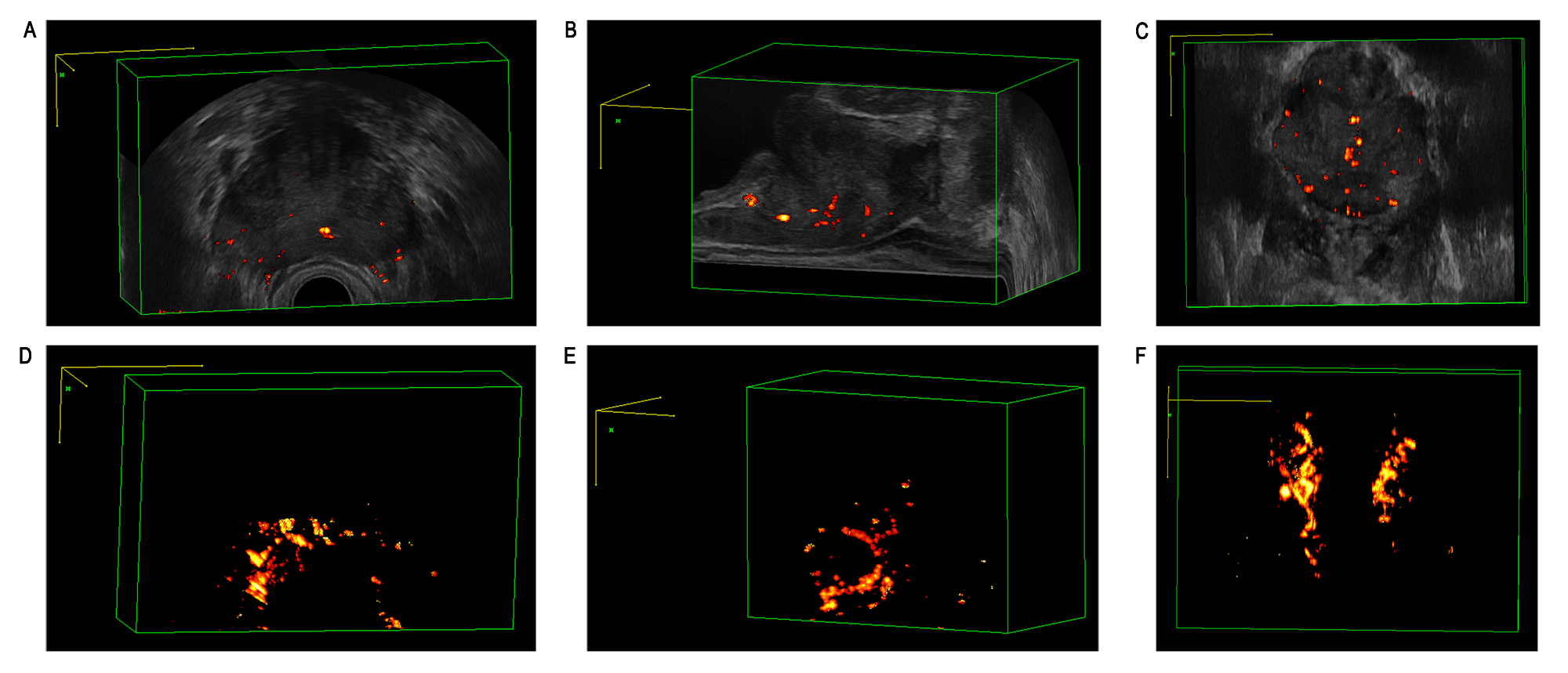

Supplement: Supplementary Figure 2 — Blood flow plethysmogram of normal person. (A) Blood flow plethysmogram of normal person in cross section; (B) Blood flow plethysmogram of normal person in longitudinal section; (C) Blood flow plethysmogram of normal person in coronal section; (D) Blood flow chart of normal person in cross section; (E) Blood flow chart of normal person in longitudinal section; (F) Blood flow chart of normal person in coronal section. [file Image_2.tiff]

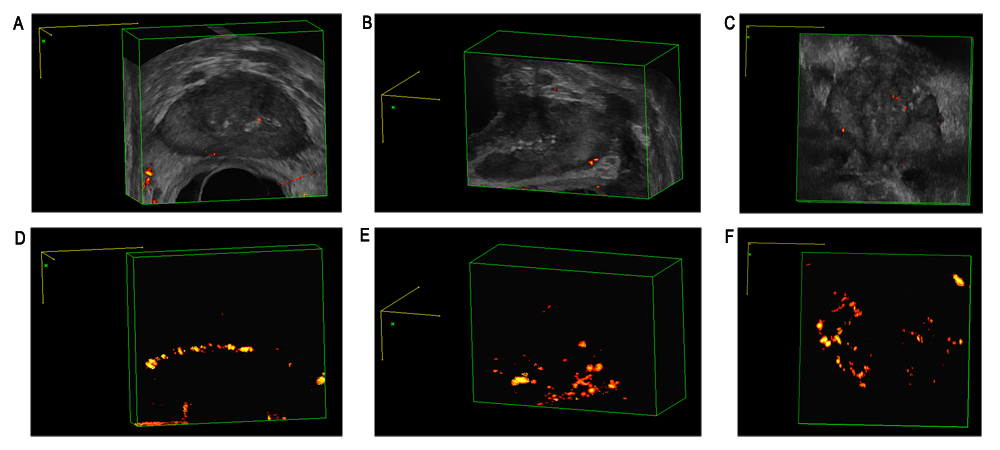

Supplement: Supplementary Figure 3 — Blood flow plethysmogram of benign prostatic hyperplasia. (A) Blood flow plethysmogram of benign prostatic hyperplasia in cross section; (B) Blood flow plethysmogram of benign prostatic hyperplasia in longitudinal section; (C) Blood flow plethysmogram of benign prostatic hyperplasia in coronal section; (D) Blood flow chart of benign prostatic hyperplasia in cross section; (E) Blood flow chart of benign prostatic hyperplasia in longitudinal section; (F) Blood flow chart of benign prostatic hyperplasia in coronal section. [file Image_3.tif]
